# Supplementary material for: Prediction of Vaccine Response and Development of a Personalized Anti-SARS-CoV-2 Vaccination Strategy in Kidney Transplant Recipients: Results from a Large Single-Center Study
Source: J Pers Med. 2022 Jul 5;12(7):1107. doi: 10.3390/jpm12071107 (PMC9317479; doi:10.3390/jpm12071107)
Supplement: Supplementary file 1 [file jpm-12-01107-s001.zip › jpm-1734844-supplementary.pdf]

Supplementary Table S1. Patient characteristics according to the antibody response after two vaccine doses (non-responders, weak responders, and moderate responders *versus* good responders)

	**Antibody titer <143 BAU (n = 437)**	**Antibody titer >143 BAU (n = 125)**	**p**
Age (years)	56.9 (±12.7)	55.9 (±14.6)	0.51
Male sex	262 (60%)	82 (66%)	0.25
Comorbidities			
BMI (kg/m²)	26.6 (±5.72)	26.7 (±5.59)	0.83
Cardiovascular disease	114 (26%)	34 (27%)	0.8
Diabetes	174 (40%)	30 (24%)	<0.01
Cancer	80 (18%)	18 (14%)	0.31
Hypertension	370 (85%)	102 (82%)	0.41
Time from kidney transplantation (years)	7.97 (±7.06)	14.5 (±9.08)	<0.001
First transplantation	363 (83%)	111 (89%)	0.12
Living donor	86 (20%)	20 (16%)	0.35
CNI			<0.001
Tacrolimus	285 (65%)	43 (34%)	
Ciclosporin	119 (27%)	65 (52%)	-
No CNI	33 (7.6%)	17 (14%)	-
Tacrolimus dose (mg)*	5.47 (±3.19)	4.75 (±2.67)	0.12
Tacrolimus trough level (µg/L)**	6.17 (±1.99)	5.71 (±1.83)	0.14
Ciclosporin dose (mg)	154 (±57.8)	146 (±51.6)	0.34
Ciclosporin trough level (µg/L)	85.0 (±39.2)	74.5 (±24.3)	0.027
MMF/MPA	370 (85%)	78 (62%)	<0.001
MMF/MPAs dose (mg)**	1262 (±525)	1046 (±491)	<0.001
mTOR inhibitors	60 (14%)	29 (23%)	0.011
mTOR inhibitors dose (mg)*	1262 (±525)	1046 (±491)	<0.001
mTOR inhibitors trough level (µg/L)	5.02 (±1.45)	5.62 (±1.67)	0.11
Azathioprine	5 (1.1%)	8 (6.4%)	<0.01
Belatacept	17 (3.9%)	1 (0.81%)	0.14
Steroids	292 (67%)	52 (42%)	<0.001
Steroids dose (mg)*	6.11 (±2.13)	5.36 (±1.86)	<0.01
Tacrolimus+MMF/MPA+steroids	177 (41%)	17 (14%)	<0.001
Serum creatinine (µmol/L)	139 (±57.6)	122 (±47.1)	<0.001
Antibody titer before the second dose ****	1.24 (±1.11)	88.8 (±459)	0.035

Data are expressed as means (± standard deviations) or counts (percentages), as appropriate. Abbreviations: BMI, body mass index; CNI, calcineurin inhibitors; MMF, mycophenolate mofetil; MPA, mycophenolic acid; mTOR, mammalian target of rapamycin.

*Missing data for one patient, **Missing data for three patients, ***Missing data for four patients, ****Missing data for two patients.

Supplementary Table S2. Patient characteristics according to the antibody response after three vaccine doses (non-responders, weak responders, and moderate responders *versus* good responders)

	**Antibody titer >143 BAU (n = 101)**	**Antibody titer <143 BAU (n = 208)**	**p**
Age (years)	56.6 (±10.9)	57.5 (±13.3)	0.5
Male sex	80 (79%)	117 (56%)	<0.001
Comorbidities			
BMI (kg/m^2)	27.6 (±5.82)	26.2 (±6.04)	0.05
Cardiovascular disease	22 (22%)	56 (27%)	0.33
Diabetes	39 (39%)	85 (41%)	0.7
Cancer	19 (19%)	42 (20%)	0.77
Hypertension	89 (88%)	176 (85%)	0.41
Time from kidney transplantation (years)	10.3 (±8.20)	6.75 (±6.14)	<0.001
First transplantation	88 (87%)	162 (78%)	0.052
Living donor	20 (20%)	43 (21%)	0.86
CNI			<0.001
Tacrolimus	49 (49%)	152 (73%)	-
Ciclosporin	45 (45%)	42 (20%)	-
No CNI	7 (6.9%)	14 (6.7%)	
Tacrolimus dose (mg)*	4.98 (±2.77)	5.76 (±3.41)	0.11
Tacrolimus trough level (µg/L)**	6.30 (±2.11)	6.16 (±1.92)	0.69
Ciclosporin dose (mg)	154 (±61.9)	164 (±56.1)	0.4
Ciclosporin trough level (µg/L)	79.3 (±52.4)	92.0 (±28.0)	0.16
MMF/MPA	84 (83%)	182 (88%)	0.3
MMF/MPAs dose (mg)*	1277 (±492)	1240 (±513)	0.58
mTOR inhibitors	12 (12%)	22 (11%)	0.73
mTOR inhibitor dose (mg)	1.83 (±0.72)	2.09 (±0.95)	0.54
mTOR inhibitor trough level (µg/L)	5.42 (±1.87)	4.81 (±1.36)	0.41
Azathioprine	2 (2%)	1 (0.48%)	0.25
Belatacept	1 (0.99%)	11 (5.3%)	0.11
Steroids	54 (53%)	159 (76%)	<0.001
Steroids dose (mg)*	6.23 (±3.21)	6.10 (±1.89)	0.77
Tacrolimus+MMF/MPA+steroids	28 (28%)	109 (52%)	<0.001
Serum creatinine (µmol/L)	127 (±44.5)	145 (±57.3)	<0.01
Antibody titer before the third dose	57.3 (±152)	2.20 (±3.5)	<0.001

Data are expressed as means (± standard deviations) or counts (percentages), as appropriate. Abbreviations: BMI, body mass index; CNI, calcineurin inhibitors; MMF, mycophenolate mofetil; MPA, mycophenolic acid; mTOR, mammalian target of rapamycin.

*Missing data for one patient, **Missing data for three patients.
